# Integrated Computing Accelerates Design and Performance Control of New Maraging Steels

**DOI:** 10.3390/ma16124273

**Published:** 2023-06-08

**Authors:** Shixing Chen, Jingchuan Zhu, Tingyao Liu, Yong Liu, Yudong Fu, Toshihiro Shimada, Guanqi Liu

**Affiliations:** 1 School of Material Science and Engineering, Harbin Institute of Technology, Harbin 150001, China; 2 State Key Laboratory of Vanadium and Titanium Resources Comprehensive Utilization, Chengdu 610300, China; liutingyao8211371@163.com; 3 College of Materials Science and Chemical Engineering, Harbin Engineering University, Harbin 150001, China; 4 Division of Applied Chemistry, Hokkaido University, Sapporo 060-8628, Japan

**Keywords:** machine learning, thermodynamic calculations, martensitic aged steel, mechanical properties

## Abstract

This paper mainly used database technology, machine learning, thermodynamic calculation, experimental verification, etc., on integrated computational materials engineering. The interaction between different alloying elements and the strengthening effect of precipitated phases were investigated mainly for martensitic ageing steels. Modelling and parameter optimization were performed by machine learning, and the highest prediction accuracy was 98.58%. We investigated the influence of composition fluctuation on performance and correlation tests to analyze the influence of elements from multiple perspectives. Furthermore, we screened out the three-component composition process parameters with composition and performance with high contrast. Thermodynamic calculations studied the effect of alloying element content on the nano-precipitation phase, Laves phase, and austenite in the material. The heat treatment process parameters of the new steel grade were also developed based on the phase diagram. A new type of martensitic ageing steel was prepared by selected vacuum arc melting. The sample with the highest overall mechanical properties had a yield strength of 1887 MPa, a tensile strength of 1907 MPa, and a hardness of 58 HRC. The sample with the highest plasticity had an elongation of 7.8%. The machine learning process for the accelerated design of new ultra-high tensile steels was found to be generalizable and reliable.

## 1. Introduction

Maraging steels are highly interesting due to their ultra-high strength [1,2,3,4,5,6,7], crucial in contemporary frontier technology fields such as aerospace, high-speed trains, deep-sea technology, advanced nuclear energy, and clean energy [8,9,10,11,12]. For example, Wuriti et al. [13] investigated the M250 maraging steel pressure vessels for aerospace applications; Dehgahi et al. [14] explored high strain-rate loadings performance of maraging steel parts in industries such as high-speed train; Jha et al. [15] researched the failure analysis of M250 maraging steel in solid propulsion system. Moreover, maraging steels are used in applications such as shielding materials in the nuclear field [16], components for thermal, wind, and hydropower generation equipment [17]. However, the limitations of this material mainly lie in the conflict between strength and plasticity. The current primary ultra-high-strength steels, such as the 18Ni system, inevitably experience a decline in plasticity as strength increases [18,19,20]. Pursuing higher overall mechanical properties was a forward-looking breakthrough direction for the future of ultra-high strength steels worldwide. With the increasing demand for lighter materials, designing and developing a new generation of ultra-high strength steels with good plasticity is urgent to achieve a breakthrough in comprehensive mechanical properties [21,22,23,24,25].

The traditional methods for strengthening martensitic steels mainly include process and composition optimization [26,27,28,29,30,31]. In process optimization, the diversity of ageing processes can significantly affect the material. For composition optimization, the influence of multiple elements on the organization and properties was widely studied. For example, Liu et al. [32] introduced nanoscale precipitation phases (M_2_C, Laves phase, α′Cr) and ductile reverse-transformation austenite by double ageing treatment and ultra-high-strength stainless steels with good plasticity and toughness were obtained by this method. Lv et al. [33] significantly reduced the content of precious elements such as molybdenum and eliminated expensive alloying elements such as cobalt and titanium, replacing them with familiar and affordable elements such as aluminium and carbon to accomplish composition optimization. In the process optimization, martensitic ageing steels with tensile strengths exceeding 2000 MPa were obtained by hot stamping and forming. To reduce the cost, Wang et al. [34] avoided precious metals such as Co and Ti. Instead, Al and a small amount of Nb were used to obtain the nanoprecipitated phase Ni (Fe, Al) and the refined NbC precipitated phase. This scheme produced martensitic steels with a tensile strength of 2100 MPa and maintained an 9.31% elongation. He et al. [35] obtained higher strengths by increasing the content of Co and Mo while keeping the Ti content extremely low to maintain the toughness. The tensile strength was 2800 MPa, and fracture toughness was 30 MPa·m^1/2^ after adjusting the alloying elements and the corresponding heat treatment.

Integrated computational materials engineering is a design development approach that integrates multi-scale computational simulations and key experiments [36,37,38]. Compared to traditional experimental investigations, this process is a significant step in developing new materials from a traditional empirical approach to a scientific design approach. As a result, the development of materials can be significantly accelerated, and the cost of development can be reduced. For example, G.B. Olson’s group at Northwestern University developed a modelling technique to fabricate Ferrium S53 through integrated materials computational engineering design. The design threshold of tensile strength of this material reached 280 ksi and was successfully applied to the landing gear of the US Air Force. This event demonstrated the feasibility of integrated materials computational engineering design [39]. Qiao et al. [40] constructed a combination-structure-property model containing physical features. A new Fe2.5Ni2.5CrAl MPEA was designed and synthesized using machine learning. Azimi et al. [41] proposed a deep learning method for microstructure classification in the example of specific microstructural compositions of mild steel. However, most current research on maraging steels focused on a single variable for a particular grade [42]. Literature-based datasets and multi-algorithmic machine-learning modelling methods have yet to be devised. Therefore, accelerating the design of new grades of maraging steels from an integrated computational perspective is an urgent problem.

This study first established a quantitative prediction model for martensitic ultra-high-strength steels to solve the problem of designing martensitic steel composition-process properties. The effect of composition fluctuations on the performance of martensitic steels was investigated by machine learning. The screened compositions were also subjected to thermodynamic calculations, an analysis of elemental influences, and process parameters. After experimental preparation and characterization, the model’s accuracy was verified to provide a database for the design of new steel grades and subsequent studies.

## 2. Materials and Methods

In this paper, a primary database of martensitic ultra-high strength steels was developed and optimized by extracting experimental data from 586 papers. The database consists of 410 data sets and 20 dimensions and contains 16 sets of the component process independent variables and four sets of performance dependent variables. The programming language used was Python, and the compiler was Jupiter. Particle swarm optimization (PSO) was used for hyperparameter optimization. The four machine learning algorithms selected were random forest (RF), support vector regression (SVR), gradient boosted regression (GBR), and bagging. Regression prediction and composition optimization of tensile strength, yield strength, elongation, and hardness of martensitic aged steel were performed. Thermo-Calc 2022a software was used for phase diagramming and investigation of the influence of composition fluctuations for the screened components, and the database selected was TCFE10.

The experimental samples were melted in an argon atmosphere using a vacuum non-self-consuming arc melting furnace. To ensure that the composition of the alloy ingots was as uniform as possible at different locations, the ingots were turned over in the crucible using a robotic arm at each melting step. This was repeated eight times to ensure the chemical homogeneity of the ingots. The ingots were cast into rectangular blocks of 150 mm × 15 mm × 15 mm using the copper mold inversion casting technique. The alloy blocks were machined by cutting using a wire-cut machine and a high-speed precision cutter (Buehler Isomet 5000, Buehler, Lexcraft, San Mateo, CA, USA) and machined into bone shaped tensile samples. Testing was performed using an X-ray diffractometer (XRD; Rigaku D/max-2500 PC, Tokyo, Japan) with scanning angles of 10° to 90° (monochromatic Cu/Kα radiation, 2 Theta). Uniaxial tensile tests were performed using an electronic universal testing machine (Instron 5582, Norwood, MA, USA) with a strain rate of 1 × 10^−3^ s^−1^. The microstructure and chemical composition of polished and electrolytically etched samples were analyzed using a scanning electron microscope (SEM, Thermo Scientific Apreo 2, Waltham, MA, USA).

## 3. Results

### 3.1. Machine Learning

**Figure 1:**
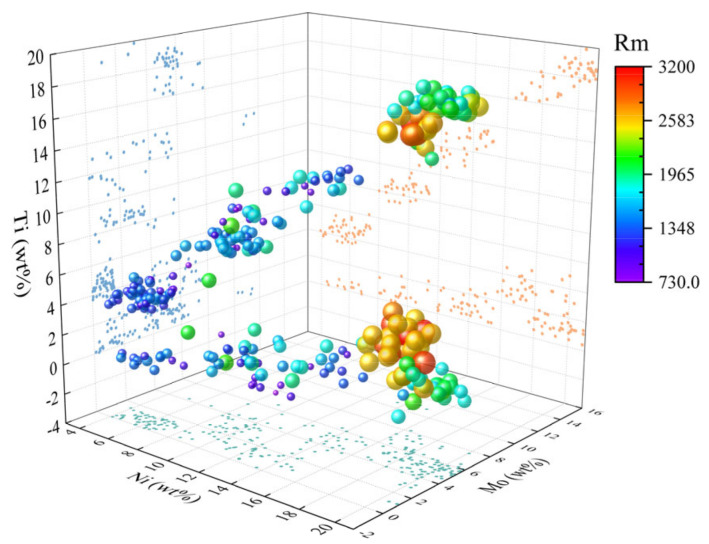
Overview of the spatial distribution of the data set.

Figure 1 shows an overview of the spatial distribution of the data set in the composition of the three elements Ti, Ni, and Mo. The colour and size of each data point represent the tensile strength value corresponding to that composition. From this figure, it can be seen that the overall tensile strength showed an increasing trend with the increase in Ni content. In addition, the data set was somewhat poorly dispersed since most of the research was focused on a few grades of steel. Therefore, the magnitude of compositional changes was limited, and the data points were concentrated. In this model, there were 12 groups of compositional independent variables. They were Ni, Co, Mo, Ti, Al, Cr, W, Cu, Nb, C, Si, and Mn. There were four groups of process variables. They were solid solution temperature, solid solution time, ageing temperature and ageing time, respectively. The model was challenging to consider comprehensively because of the many factors affecting the properties in real situations. The prediction results were difficult to achieve complete accuracy.

**Figure 2:**
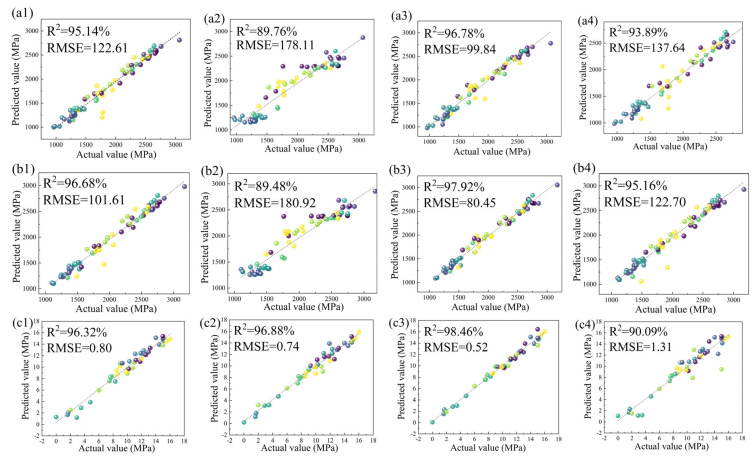

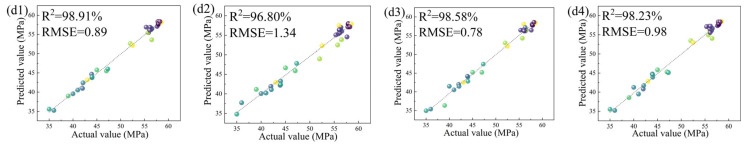
The regression analysis of the four algorithms’ training effects, RF, SVR, GBR, and BR. (**a1**–**a4**) Rp; (**b1**–**b4**) Rm; (**c1**–**c4**) A; (**d1**–**d4**) Hv.

Figure 2 shows the regression analysis of the training effects of the four algorithms, RF, SVR, GBR, and BR. To quantitatively assess the accuracy of each model for the subsequent design of new steel types, R^2^ and RSME were calculated for each data point, as shown in the top left corner of each small plot in Figure 2. Generally speaking, the closer the R^2^ value was to 1, the better the fit was. Of the four algorithms, the GBR algorithm had the highest R^2^ of 98.58% for the hardness model. Its RSME was 0.78. Compared with the other algorithms, the GBR algorithm had the highest R^2^ for all of them; so, the model had a significant advantage in designing new steel grades for maraging steel, with the best generalization capability and the highest prediction accuracy. The model will also be used in subsequent predictions for the whole composition space and the effect of composition fluctuations on performance analysis.

**Figure 3:**
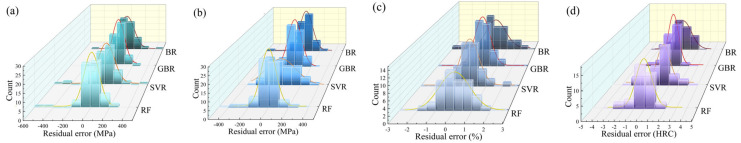
Residual Regression Fitting of Four Algorithms. (**a**) Rp; (**b**) Rm; (**c**) A; (**d**) Hv.

Figure 3 shows the residual plots and regression fitting curves for the four algorithms for 16 models. Based on the regression fitting curves for the random errors, it can be seen that of the four algorithms, the GBR algorithm had residuals that were most normally distributed. Most of the sample data were concentrated around the mean and were in the overall majority. The distribution of the residuals of the other algorithms did not conform to a normal distribution enough, such as the SVR algorithm in Figure 3b and the RF and BR algorithms in Figure 3c. The residuals need to obey a normal distribution. Therefore, these three models were not ideal enough for fitting the mechanical properties of maraging steel.

The analysis of the four algorithms showed that bagging handled high-dimensional data well and reduced overfitting by combining multiple models. RF was also resistant to overfitting due to built-in bagging. Meanwhile, it handled high-dimensional data very efficiently because of combining the predictions from multiple decision trees. Compared to RF, SVR used a kernel function to handle non-linear data, and so, it performed well when the number of features was high. As for GBR, it worked by iteratively training weak decision trees and combining them to create a strong learner. So, GBR handled non-linear data well. However, SVR and GBR were prone to overfitting if parameters were not tuned correctly. and the training took more time due to the large amount of computation.

Compared to the other algorithms, the GBR algorithm, although more time-consuming, was more suitable for application in this maraging steel field to complex non-linear problems and can achieve optimum accuracy and generalization capability.

**Figure 4:**
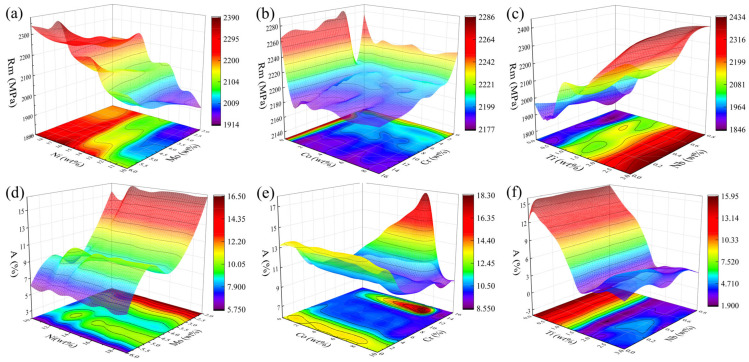
Element-performance impact trend Prediction Chart. (**a**) Ni–Mo–Rm; (**b**) Co–Cr–Rm; (**c**) Ti–Nb–Rm; (**d**) Ni–Mo–A; (**e**) Co–Cr–A; (**f**) Ti–Nb–A.

Figure 4 shows the predicted trend of element-property effects. It mainly included the predicted trends of the effects of Ni–Mo, Co–Cr, and Ti–Nb on tensile strength and elongation. From Figure 4a,d, it can be seen that with the increase in Ni content, there was a significant increase in tensile strength, but the change of elongation was not apparent; with the increase in Mo content, the rise of tensile strength was not high, but the elongation decreased significantly. The analysis of Figure 4b,e shows that the tensile strength decreased slightly with the increase in Co and Cr content, but generally, they were above 2100 MPa. The effect of Co content on the increase in elongation was less obvious, and the trend of elongation decreased and then increased with the increase in Cr content. The specific influence trend needs to be analyzed, subsequently, concerning the correlation coefficients. In Figure 4c,f, it can be seen that the tensile strength gradually increased, and the elongation decreased significantly with the increase in Ti content, while the Nb content was not sensitive to the elongation.

Table 1 shows the correlation test results for the four mechanical properties. The table selects the five factors with the highest absolute correlation value to the mechanical properties for display. In terms of yield strength and tensile strength combined, the elements positively correlated with the strength of the maraging steel were Ni, Mo, Co, and Ti, and negatively correlated with the strength were Cr, Cu, and Nb. The results of this correlation coefficient calculation were only affected by the maraging steel data set in this paper, and if the physical and process conditions change under other conditions, the role of the elements will also change. The correlation test results for hardness were similar to those for strength, and the elements with the highest correlations were Mo and Co elements, both of which had positive correlations. In contrast, Cr, Nb, and Cu played a role in reducing hardness. Slightly different from hardness and strength, among the top five elements with absolute values of correlation coefficients, only the Cr element was positively correlated with elongation, while Mo, Co, Ni, and Ti were all negatively correlated.

Table 2 shows the three sets of compositions with high-performance differences after being screened by machine learning modelling. The purpose of screening these three sets of compositions was mainly twofold. First, since there were 16 groups of independent variables in the original model, it was difficult to distinguish the specific factors causing the performance differences if all 16 variables were set to change simultaneously. In addition, since there were fewer studies on additional elements such as Cu and Nb in practical work, the collected data could be more reliable in the model. Therefore, in this paper, only five elements commonly found in martensitic ageing steels, Ni, Co, Mo, Ti, and Al, were selected and screened for combinations with significantly different compositional and performance gradients. This aimed to investigate better the effect of elements on properties and the accuracy of the machine learning model. The foundation for future model optimization and multi-element experimental investigations is laid.

### 3.2. Thermodynamic Calculations

**Figure 5:**
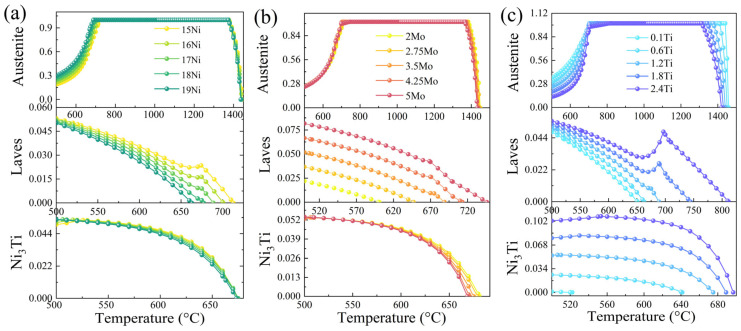
Effect of Ni, Mo, Ti on austenite, Laves phase, and Ni_3_Ti by thermodynamic calculation (with 12.5Co−0.1Al). (**a**) xNi−3.5Mo−1.2Ti, (**b**) 17Ni−xMo−1.2Ti, (**c**) 17Ni−3.5Mo−x1.2Ti.

Figure 5 analyzes the impacts of the changes in the content of Ni, Mo, and Ti on austenite, laves phase, and Ni_3_Ti. From Figure 5a, it can be seen that the austenite content tended to increase to 700 °C as the Ni content rose. It shows a decreasing trend after about 1350 °C, but the difference was less noticeable. For the analysis of the Laves phase and Ni_3_Ti phase content, as the Ni content gradually increased, the Laves phase and Ni_3_Ti phase appeared at a lower temperature, and the content gradually decreased. From Figure 5b, it can be seen that the change of the austenite phase was not evident with the increase in Mo content; the Laves phase increased with the increase in Mo content and the appearance temperature increased; the appearance temperature of Ni_3_Ti phase decreased. From Figure 5c, it can be seen that the austenite amount decreased significantly as the Ti content increased; the Laves phase and Ni_3_Ti content increased significantly and the emergence temperature increased significantly.

**Figure 6:**
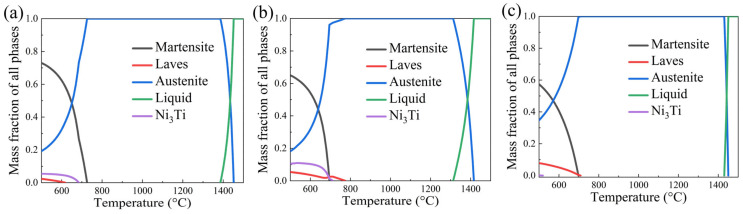
Thermodynamic calculation of the phase diagram of samples No. 1–3 (with 12.5Co−0.1Al). (**a**) 15Ni−2Mo−1.2Ti, (**b**) 19Ni−3.5Mo−2.4Ti, (**c**) 17Ni−5Mo−0.1Ti.

In order to design process parameters for the screened three-component compositions, the Thermo-calc software was used in this paper to calculate the phase diagrams of samples 1–3 at 500–1500 °C, as shown in Figure 6. The composition in Figure 6b had the highest Ni_3_Ti precipitated phase content, about 10% at 500 °C. The lowest Ni_3_Ti content was in the composition corresponding to Figure 6c—all the precipitated phases dissolved above 800 °C. In order to dissolve the precipitated phases and keep the grains from growing drastically, the solid solution process was set to 820 °C for one h and oil cooling. The ageing process was set to 480 °C for three hours and air-cooled by combining the machine learning parameter prediction, phase diagram, and reference to the conventional martensitic ageing process. In order to improve the strength of the material and inhibit grain growth, the material was deep cooled in the middle of the solid solution and ageing steps, which was −73 °C liquid nitrogen treatment for one hour + air cooling.

**Figure 7:**
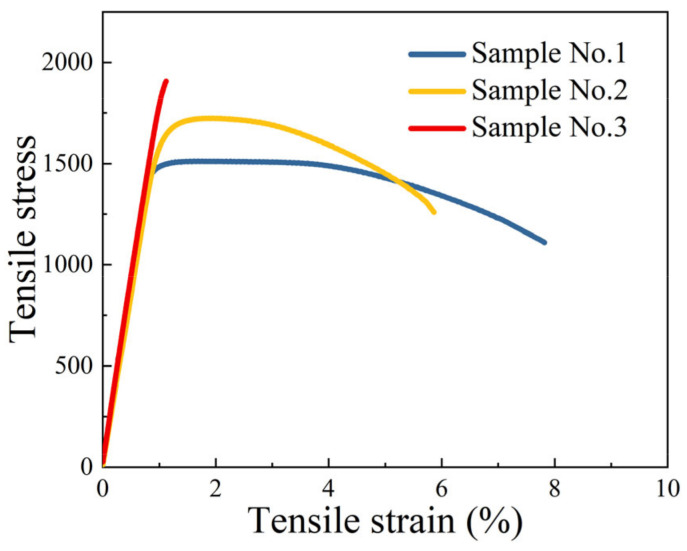
The stress–strain tensile curves of 3 samples.

Figure 7 shows the tensile test graphs of the three sets of samples. From Figure 7, it can be seen that the highest elongation of 7.82% was obtained for sample No. 1, 5.86% for sample No. 2, and 1.12% for sample No. 3. This coincides with the analysis from Figure 4 and Table 2. The elongation decreased significantly as the Mo content increased. The highest tensile strength among the three groups of samples was sample No. 3, which also had the highest Mo and Ni content. This coincided with the previous analysis of the effect of Mo and Ni elements.

Table 3 shows the list of actual compositions and actual values of mechanical properties. Among the three groups of samples, the highest hardness was found in sample No. 2, with a value of 58.7 HRC, which confirmed the positive correlation between Mo and Co content and hardness in the correlation analysis. In Table 3, comparing the experimental values with those reported in the literature, it can be seen that the performance of sample No. 3 prepared in this paper was more outstanding. The remaining two samples did not differ significantly from the literature. However, the new steel developed in this experiment differed significantly from the literature composition. The complexity of the variables made it difficult to analyze the effects brought about by a particular element individually.

Comparing the predicted values with the actual values in Table 2 and Table 3, it can be seen that the errors in terms of tensile strength, yield strength and hardness were less than 10%. In terms of elongation, the error between the two was very high. This result was due to the addition of the deep cooling step compared to the solid solution + ageing process in the data set. This step led to grain refinement, increased strength, and reduced the material’s plasticity. To conclude, the machine learning model in this paper was generally consistent with the actual situation and had some generalization capability and accuracy.

**Figure 8:**
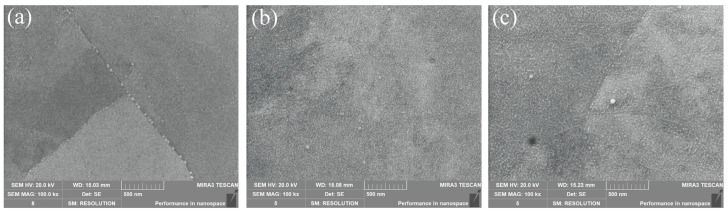
Surface morphology SEM images of samples No. 1–3. (**a**) Sample No. 1; (**b**) Sample No. 2; (**c**) Sample No. 3.

Figure 8 shows the surface SEM images of the samples. Overall, the surface of these samples was almost free of defects. In Figure 8a, the dark grey matrix was the martensitic phase with fine white Ni_3_Ti nanophases uniformly distributed on the grain boundaries. Compared with Figure 8a, the nanoscale Ni_3_Ti particle phase in Figure 8b was distributed within the grain, whereas the nanophase in Figure 8c was distributed at the grain boundaries as well as within the grain. This phenomenon explains the reason why sample No. 3 possessed the highest hardness. When the nanophase was present at the grain boundary and inside the grain, the strengthening effect occurred in the grain itself, at the interface between the matrix phase and the precipitated phase, and between the matrix phases; this was so that Sample No. 3 was less prone to deformation and damage when subjected to the pressure of the indenter.

**Figure 9:**
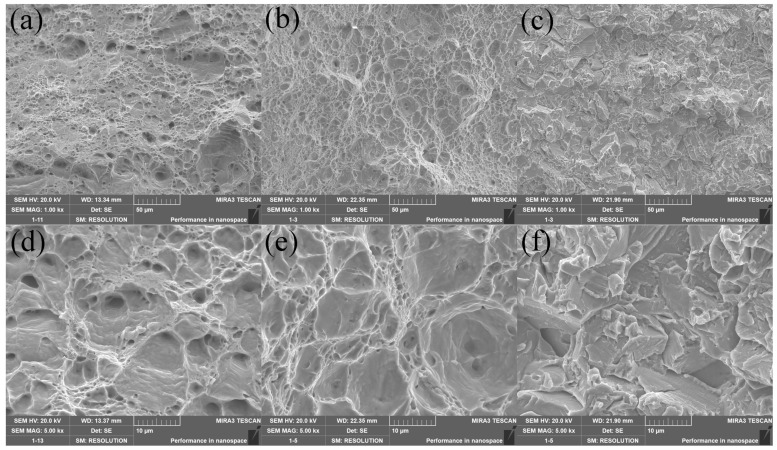
Fracture morphology SEM images of samples No. 1–3 with 1000× and 5000×. (**a**,**d**) Sample No. 1; (**b**,**e**) Sample No. 2; (**c**,**f**) Sample No. 3.

Figure 9 shows the fracture morphology of the prepared samples. Figure 9a,b show microporous aggregation-type fractures, and Figure 9c shows quasi-dissolution fractures or brittle fractures. In Figure 9a, the micropore size was more prominent and more profound, and the dimples pattern was evident. In contrast, Figure 9b also had a more apparent dimples pattern, but the micropore size was smaller and dense, and the micropores were shallow. Therefore, it can be presumed that the toughness of Figure 9b was less than that of the sample in Figure 9a. In Figure 9c, it can be seen that the fracture mode of this sample was a brittle fracture, and the primary fracture mechanism was a mixed fracture mode of transgranular fracture and intergranular fracture. The river-like pattern of transgranular fracture and the grain pull-out trace of along-crystal fracture can be seen in the figure. Therefore, it can be speculated that this sample had the highest strength and lowest plasticity among the three samples. Combined with Table 3, it can be seen that the speculation entirely agreed with reality.

In summary, the mechanical properties of the samples prepared in this paper were in substantial agreement with the results predicted by the machine learning model, while the analysis for this model also explained the effect of each element on the properties. This process of multi-algorithm modelling, composition screening, discussion of influencing factors, thermodynamic calculations, and experimental validation was accurate and repeatable. Additional elemental influences can be explored for maraging steels based on this study, and descriptors can be incorporated to improve the generalizability and accuracy of the models. It can even be used to develop other new steel grades with considerable development potential.

## 4. Discussion

(1) The GBR algorithm had the highest prediction accuracy, with R^2^ of 96.78%, 97.92%, 98.46%, and 98.58% for yield strength, tensile strength, elongation, and hardness, respectively. The correlation coefficients and three-dimensional prediction diagrams showed that Ni, Mo, Ti, and Co elements were positively correlated with the strength and negatively correlated with the elongation of the maraging steel; among them, Mo and Co positively affected the hardness.
(2) Thermodynamic calculations showed that with the increase in Ni and Mo content, the temperature of the Ni_3_Ti precipitation phase decreased, and the content gradually decreased; with the increase in Ti content, the equilibrium temperature and content of Ni_3_Ti precipitation phase showed an increasing trend.
(3) The matrix organization of the samples after ageing was martensite. The martensitic grain boundaries and intercrystalline fine dispersed nanoscale particles precipitation phase were mainly composed of Ni_3_Ti. The fracture mechanism of the samples with higher plasticity was mainly microporous polymerization fracture, while the samples with lower plasticity were mainly composed of quasi-cleavage or brittle fracture.
(4) The experimentally prepared 17Ni-12.5Co-5Mo-0.1Ti new martensitic aged steel had a tensile strength of 1907 MPa, an elongation of 1.12%, and a hardness of 58 HRC. The error between this and the predicted result was within 10%, which verified the model’s accuracy and provided a research idea with high generalization capability and high efficiency for machine learning to accelerate the design of new ultra-high-strength steels.

## 5. Conclusions

In this paper, based mainly on the basic framework and technical means of integrated computational materials engineering, machine learning, thermodynamic calculations, and experimental validation were applied to develop a highly accurate and scalable means of designing new steel grades. In the future, first-principles calculations and Monte Carlo simulations, combined with various characterization tools such as APT and TEM, will also be used to systematically explain the micromechanical behavior mechanism at the atomic scale. In addition, there are still great prospects for exploring metal forming processes, such as rolling and forging of this material.

## Figures and Tables

**Table 1:** The top five absolute values of the independent variables in the correlation test.

Rp_0.2_		Rm		A		Hv	
Ni	0.766	Co	0.857	Mo	−0.742	Mo	0.936
Mo	0.700	Mo	0.770	Co	−0.718	Co	0.933
Cr	−0.699	Cr	−0.717	Cr	0.601	Cr	−0.852
Co	0.658	Ni	0.632	Ni	−0.440	Nb	−0.686
Ti	0.611	Cu	−0.470	Ti	−0.314	Cu	−0.652

**Table 2:** A list of the composition and process of the new maraging steel designed in this paper.

No.	Ni	Co	Mo	Ti	Al	Fe	Rp-Pre	Rm-Pre	A-Pre	Hv-Pre
1	15	12.5	2	1.2	0.1	Bal.	1442	1496	9.14	53.8
2	19	12.5	3.5	2.4	0.1	Bal.	1609	1672	6.56	52.7
3	17	12.5	5	0.1	0.1	Bal.	1754	1811	3.54	59.2

**Table 3:** The composition and performance of maraging steel in this paper and reported values from the literature [43,44,45].

No.	Ni	Co	Mo	Ti	Al	Fe	Rp-act	Rm-act	A-act	Hv-act
1	14.89	12.45	2.11	1.21	0.09	Bal.	1487	1512	7.82	54.2
2	19.02	12.47	3.24	2.21	0.06	Bal.	1694	1724	5.86	51.5
3	16.87	12.43	4.96	0.13	0.08	Bal.	1887	1907	1.12	58.7
4 [43]	18.72	8.94	5	0.92	0.12	Bal.	1634	1798	4.8	52.3
5 [44]	8.3	10.56	5.51	0.2	0.41	Bal.	1576	1602	8.1	-
6 [45]	11.14	0	0.016	1.65	0.154	Bal.	1609	1637	2	-

## Data Availability

Restrictions apply to the availability of these data. Data were obtained from Shixing Chen and are available with the permission of Shixing Chen.
